# Effect of Different MPTP Administration Intervals on Mouse Models of Parkinson's Disease

**DOI:** 10.1155/2022/2112146

**Published:** 2022-03-02

**Authors:** Yuanyuan Ma, Qiongwen Rong

**Affiliations:** ^1^Department of Neurology, The First Affiliated Hospital of Hainan Medical University, Haikou 570102, China; ^2^Hainan Medical University, Haikou 571199, China; ^3^Key Laboratory of Brain Science Research and Transformation in Tropical Environment of Hainan Province, Haikou 571199, China

## Abstract

**Objective:**

To research the effect of different 1-methyl-4-phenyl-1,2,3,6-tetrahydropyridine (MPTP) administration intervals on the behavior and pathology of mouse models of Parkinson's disease.

**Methods:**

Eighteen C57 male mice were divided into a control group, subacute model group, and chronic model group (6 mice per group). Animal models of Parkinson's disease were built according to MPTP administration. The behavior of mice was determined through an open-field test and pole test. Tyrosine hydroxylase expression in brain tissues was checked by immunohistochemistry and western blot.

**Result:**

In the open-field test, the total activity distance in the chronic model group (1271.05 ± 207.93 cm) was reduced significantly compared with that of the control group (1964.21 ± 379.77 cm), while the distance had no significant differences in the subacute model group (1950.57 ± 273.54 cm). At the same time, the number of times the mice crossed the center grid in the chronic model group (3.17 ± 1.17) was reduced compared with that in the control group (11.67 ± 6.65), while there were few differences in the subacute model group (9.33 ± 2.81). In the pole test, the climbing time (8.49 ± 1.44 s) and total rest time (103.64 ± 26.57 s) of mice in the chronic model group were longer than those in the control group, respectively (4.31 ± 0.70 s, 45.21 ± 14.36 s), while there were no significant differences in the subacute model group (4.51 ± 0.48 s, 52.44 ± 25.98 s). Besides, compared with the control group, TH expression in the subacute model group and chronic model group was reduced notably, and the changes of TH expression in the chronic model group were more significant.

**Conclusion:**

There is a little loss of midbrain dopaminergic neurons in the subacute Parkinson's disease mouse models induced by continuous MPTP administration, but there is no effect on the behavior. Long interval MPTP-induced chronic Parkinson's disease mouse models lose a lot of dopaminergic neurons, which is accompanied by anxiety-like behaviors in addition to motor dysfunction.

## 1. Introduction

Parkinson's disease (PD) is one of the most common neurodegenerative disorders. In 2021, PD affected about 1.5% of population over 65 years of age worldwide, and its incidence is increasing [1]. PD is mainly characterized by somatomotor system dysfunction (rigid muscles, slowing of body movements, impaired posture and balance, difficulty with walking, and shaking or tremor). The cores of PD are the degeneration of nigrostriatal dopaminergic pathways, substantial loss of substantia nigra pars compacta (SNpc) neurons, and the depletion of dopamine (DA) [2–4]. Tyrosine hydroxylase (TH) is an important rate-limiting enzyme for DA synthesis in the substantia nigra of the brain, and its changes are positively correlated with DA changes [5]. In addition to typical motor symptoms, most of PD patients are accompanied with nonmotor symptoms such as anxiety and dyssomnia [6]. Current treatment options are mostly to relieve symptoms with anticholinergic drugs, which are not appropriate for the patients with cognitive impairment and those with renal impairment [7]. At present, there is no neuroprotective therapy to treat or stop the progression of PD other than to relieve some related symptoms [8]. The mechanism of action to effectively prevent the progression of PD is still unknown [9]. Therefore, the mechanism of PD is still the focus of future research.

**Figure 1:**
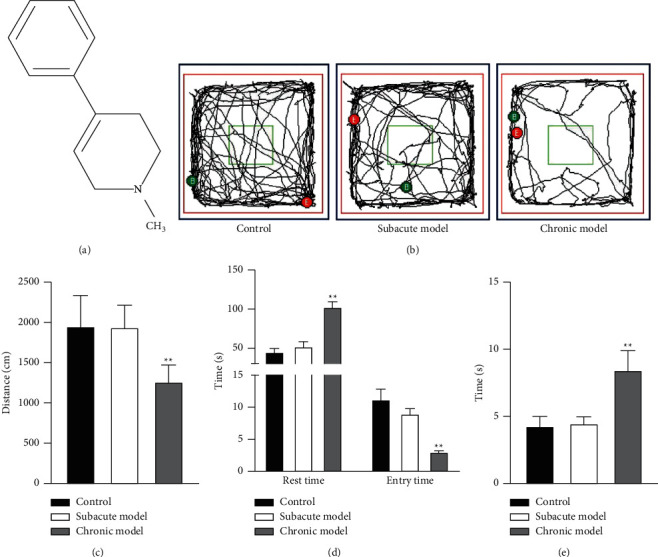
Effects of different modes of MPTP administration on motor function and anxiety behavior in mice. (a) Chemical structure of 1-methyl-4-phenyl-1,2,3,6-tetrahydropyridine (MPTP). (b) Behavior trajectory diagram of mice in the open-field test: red is the mouse activity outer frame line and green is the center grid, B represented start position, and E represented the end of recording the mouse position. The open-field test was utilized to check the total movement distance (c) and the total rest time (d) of the mice in each group. (e) Pole test was applied to determine the motor function of mice in each group. ∗∗*P* < 0.01 vs. control group.

1-Methyl-4-phenyl-1,2,3,6-tetrahydropyridine (MPTP) (Figure 1(a)) is a lipophilic neurotoxin, which can cross the blood-brain barrier and induce PD [9]. The neurotoxic drug MPTP-induced PD model is still the mainstream animal model because it is easy to establish and can mimic the motor and nonmotor symptoms of PD in humans [10]. On the basis of different establishment methods, mouse models of MPTP-induced PD can be classified into an acute or a subacute model and chronic model [11]. MPTP mouse models are mainly applied in the acute or subacute model because both injury and recovery occur faster in this model. The MPTP-induced chronic PD mouse model is characterized by a slow decrease and low-level maintenance of striatal DA content, which is more consistent with the features of the chronic progressive course of human PD. However, there are currently few comparative studies on the two models [12]. Therefore, in this study, MPTP-induced subacute and chronic PD mouse models were studied in terms of motor symptoms, nonmotor symptoms, and the degree of dopaminergic neuroreduction, providing a suitable disease model for subsequent molecular mechanism research and drug development in the pathogenesis of PD.

## 2. Materials and Methods

### 2.1. Experimental Animals

Eighteen SPF male mice (age: 8 weeks; weight: 20.73 g ± 0.93 g) were offered by Topgene Biotechnology Co., Ltd. (Changsha, China). The mice were housed in ambient temperature (20–24°C) with a relative humidity of 55–60% and 12 h light/dark cycles. The mice were fed ad libitum for one week to conduct subsequent experiments.

### 2.2. Establishment of Subacute and Chronic PD Models

Eighteen C57 male mice were divided randomly into three groups (6 mice per group): control group: intraperitoneal injection of 0.2 mL of normal saline was performed twice a week for 5 consecutive weeks; subacute model group: intraperitoneal injection of MPTP (30 mg/kg; Sigma, USA) was performed once a day for 10 consecutive days; chronic model group: intraperitoneal injection of MPTP (30 mg/kg) was performed once 3.5 days for 5 consecutive weeks. MPTP administration was performed 10 times in each group. Brain tissues were extracted after the mice were sacrificed [13].

### 2.3. Open-Field Test [14]

Mice were placed individually in the center of an open field composed of white acrylic panels (450 mm × 450 mm × 400 mm). A camera was placed 1 m directly above the activity field of the mice. Each mouse was moved within the open field for 5 min after initiation. The odor was eliminated with 75% ethanol and clean water after each mouse finished the test. Smart 3.0 video tracking software was applied for the analysis of total activity distance, number of times across the center lattice, and distance of mice.

### 2.4. Pole Test [15]

A foam ball with a diameter of 2.5 cm was placed on the top of a self-made pole with a length of 50 cm and diameter of 1 cm. The surface of the pole was wrapped with gauze. The mice were trained for 3 days before the start of the formal experiment. Mice were placed with their heads up on top of the ball, and the time for mice to touch the platform with both forelimbs was recorded. The trial was repeated three times and the average value was recorded. Each experimental interval was more than 1 min, and the residual odor was removed with alcohol.

### 2.5. Western Blot

Brain tissue was lysed on ice by RIPA protein lysate. The tissue was centrifuged at 12000 rpm at 4°C for 15 min, and then the supernatant protein was collected. After the concentration of protein was detected using a BCA protein assay kit (ThermoFisher), loading buffer was added at the proportion of 4 : 1. Furthermore, the protein was boiled for 3–5 min. After SDS-PAGE gel electrophoresis, the protein was transferred onto a PVDF membrane. Subsequently, 5% skimmed milk was added for one-hour sealing. Then, the protein was incubated with TH antibodies (Abcom, USA) at 4°C overnight. Furthermore, the second antibodies were added for 2-hour incubation at ambient temperature. Then, the protein was washed 3 times using TBST. Exposer was adopted to develop. Image J was utilized to analyze the gray value.

### 2.6. Immunohistochemistry

After mouse brain tissues were fixed for 48 h with 4% paraformaldehyde, wax blocks were prepared. Then, the tissues were sliced into 6 *μ*m sections. After dewaxing and rehydration, antigen retrieval, serum blocking, and other steps, anti-TH antibodies were added for incubation at 4°C overnight. Then, the secondary antibodies were added for one-hour incubation at ambient temperature. Diaminobenzidine (DAB) was applied for color development and hematoxylin was added for redyeing. After gradient dehydration with alcohol and clearing with xylene, the neutral resin was utilized for mounting. Then, the tissues were observed and photographed under a microscope. Image J was performed for counting TH-positive neurons.

### 2.7. Statistical Analysis

All data were analyzed using SPSS 22.0 software. Mean ± standard deviation (mean ± SD) was adopted to present the data. A one-way analysis of variance was selected for the data statistics between multiple groups, and *t*-test was selected for the comparison between two groups. *P* < 0.05 was regarded to be statistically significant.

## 3. Result

### 3.1. Different Modes of MPTP Administration Cause Differences in Motor Function and Anxiety Behavior in Mice

The motor functions of mice in models were compared by an open-field test and a pole test. The result revealed that, in an open-field test, the mice in the subacute model group and control group moved more in the central grid and their movements were more active, while the movements of mice in the chronic model group were fewer (Figure 1(b)). Besides, the total distance of spontaneous activity of mice in the chronic model group (1271.05 ± 207.93 cm) after the MPTP intervention was significantly reduced compared with that in control groups (1964.21 ± 379.77 cm), while there were few differences between the subacute model group (1950.57 ± 273.54 cm) and the control group (Figure 1(c), *P* < 0.05). Also, the open-field test could reflect the anxiety behavior of mice. The result disclosed that the number of times the mice crossed the center grid in the chronic model group (3.17 ± 1.17) declined significantly compared with that in the control group (11.67 ± 6.65), while there were few differences in the subacute model group (9.33 ± 2.81). At the same time, both the climbing time (8.49 ± 1.44 s) and total rest time (103.64 ± 26.57 s) of mice in the chronic model group were longer than those in the control group (4.31 ± 0.70 s, 45.21 ± 14.36 s), while there were no significant differences in the subacute model group (4.51 ± 0.48 s, 52.44 ± 25.98 s) compared with the control group (Figures 1(d) and 1(e), *P* < 0.05).

### 3.2. Different Modes of MPTP Administration Cause Differences in the Degree of Dopaminergic Neuron Reduction in the Substantia Nigra of the Brain in Mice

**Figure 2:**
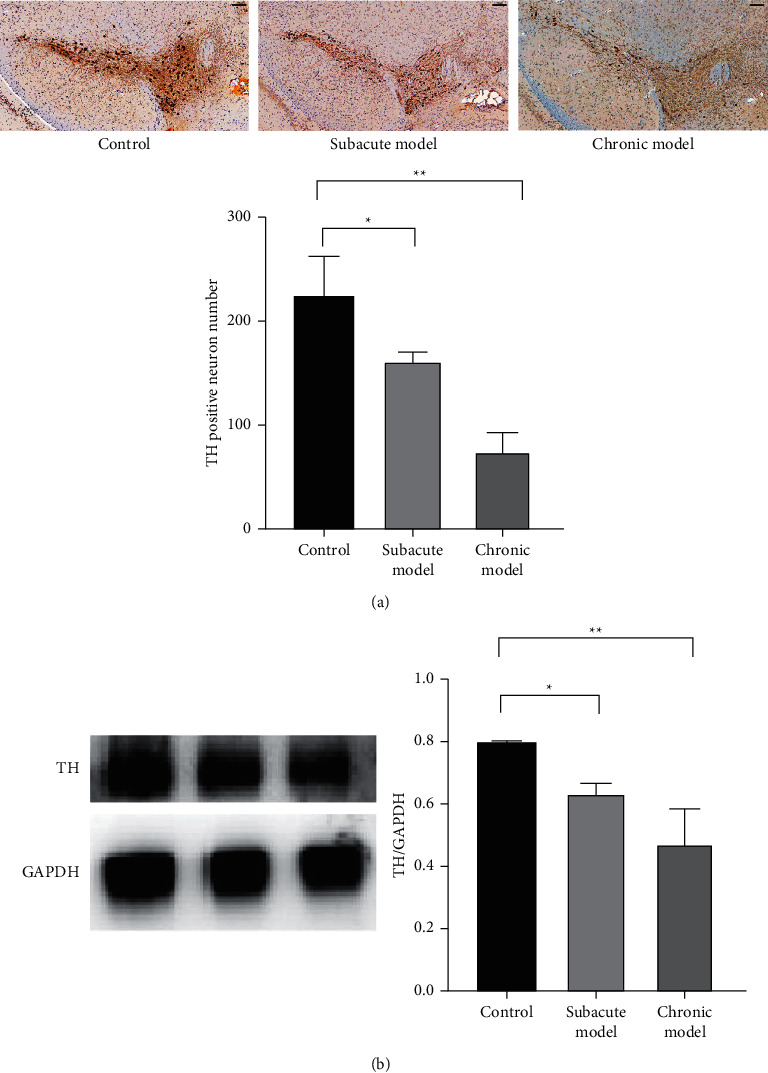
Effects of different modes of MPTP administration on the degree of dopaminergic neuron reduction in the substantia nigra of the brain in mice. Immunohistochemistry (x100, scale bar = 100 *μ*m) (a) and western blot (b) were applied to determine tyrosine hydroxylase expression in mouse brain tissues. ∗*P* < 0.05, ∗∗*P* < 0.01 vs. control group.

TH, the rate-limiting enzyme in the biosynthesis of DA, was a marker of dopaminergic neuronal. Immunohistochemistry and western blot were adopted to compare the level of TH in the brain tissues of mice in each group. The results revealed that, compared with the control group, TH-positive cells in the subacute model group and chronic model group reduced notably (Figures 2(a) and 2(b), *P* < 0.05).

## 4. Discussion

PD is a complex progressive neurodegenerative disorder. The pathological markers of PD included motor symptoms such as the loss of nigrostriatal dopaminergic neurons, occurrence of Lewy bodies, striatal dopaminergic denervation, static tremor, postural disorder, muscle rigidity, and nonmotor symptoms such as anxiety, constipation, hyposmell, sleep deprivation, and pain [16, 17]. Now, levodopa and surgery remain the main methods for the treatment of PD. However, surgery is just effective for some specific patients and its success rate is approximately 8–10%, and the drug treatment is mainly to improve motor symptoms [18]. After more than 200 years of exploration, the pathogenesis and molecular mechanism of PD have not been completely clarified, and the therapeutics that can fully relieve the symptoms of PD still remain unknown [19]. Therefore, there is a need for a simple and available animal alternative model that can mimic the pathology and behavior of human PD, thereby better studying the pathogenesis and new treatments of PD.

MPTP, a simple pyridine compound, is a by-product of heroin, which can induce PD in humans [20]. The administration of MPTP in the development of PD animal models exerts a significant function not only in the discovery of new therapies for the treatment of motor symptoms in PD but also in the search for potential causes of the disease [21, 22]. MPTP-induced PD models are divided into disease models with different disease courses according to different doses, times, intervals, and durations of administration. However, the uniform standard dose and time are still unsure. Machado et al. pointed out that disease models could be divided into the following: acute model: MPTP (20 mg/kg) was administered at a two-hour interval for a total of 4 times; subacute model: MPTP (20 mg/kg–30 mg/kg) was administered once a day for 5 consecutive days; progressive chronic model: MPTP (4 mg/kg) was administered once a day for over 20 days [23]. The study by Zhang et al. found that the subacute model could be established according to the following method: MPTP (30 mg/kg) was administered once a day for 5 consecutive days [14]. Lai et al. applied a chronic progressive model: MPTP (18 mg/kg) was administered twice a week for 5 consecutive weeks [24]. Martín-Montañez et al. also built a chronic progressive model: MPTP (24 mg/kg) was administered twice a week for 5 consecutive weeks [25]. Conventionally, it can be an animal model for PD mice as long as the dopaminergic neuron loss in the substantia nigra of the midbrain treated by MPTP is statistically different compared with normal mice. In this study, from motor symptoms, nonmotor symptoms, and pathology, the pole test [26]and open-field test [27] were applied to investigate the comparisons between different PD models established according to different MPTP administration intervals. The results disclosed that, in pole test, mice in the chronic model group had a prolonged climbing time compared with the control group, while the climbing time of those in the subacute model group had no significant change. In the open-field test, the total activity distance of mice in the chronic model group was shortened, while there were no obvious changes in the subacute model group. The result above revealed that there was no significant change for the motor function of subacute model mice, while chronic model mice showed hypokinesia. Open-field test can reflect anxiety-like behavior in animals [27]. In this study, anxiety behavior occurred in mice in the chronic model group. Besides, after MPTP administration, the rest periods of mice were prolonged in varying degrees. The total rest time of mice in the chronic model group was prolonged, while there was no significant difference between the subacute group and control group. The total rest time prolonged in open-field test also confirmed from side that the locomotion and spontaneous exploration ability of mice were declined. The result above is consistent with previous reports, suggesting that the chronic PD model has the nonmotor symptoms of anxiety in PD. The chronic PD model can better imitate the function and nonmotor symptoms of human PD and can reflect the characteristics of the disease. It is an important animal disease model applied to explore the pathogenesis of PD and screen therapeutic drugs [28, 29].

The number of dopaminergic neurons in the midbrain is closely related to the condition of PD. TH is the rate-limiting enzyme in the synthesis of DA, and TH positive neurons can represent dopaminergic neurons. In this study, after MPTP administration, the number of TH positive cells was significantly decreased in mice, and the decrease was more pronounced in chronic model mice. At the same dose, chronic MPTP administration had a more significant effect on midbrain dopaminergic neurons in mice. Under the condition of chronic MPTP administration, the mice showed hypokinesia and developed anxiety-like behavior, which was more similar to the performance of human PD. In summary, chronic models can better simulate the motor and nonmotor symptoms of human PD, providing an ideal animal model for studying the pathogenesis and finding new pathways of PD. Chronic models are worthy of promotion for the study of PD.

It is worth mentioning that during the course of trial, after MPTP administration, all mice showed piloerection and decreased locomotion. In the chronic model, after MPTP administration for 4-5 times, mice were injected intraperitoneally with MPTP. About five minutes later, mice showed different degrees of nonstop shaking of both upper limbs on the head and tetanic twitching of the tails, which could improve spontaneously after several minutes. However, in the subacute model, there were no similar phenomena. The result above indicated that the systemic effects of chronic MPTP administration on mice were more significant. However, the mechanism of the effect of MPTP administration on mice in subacute and chronic PD models is not yet clear, which requires further research.

## 5. Conclusion

To sum up, MPTP-induced subacute PD model mice showed a small amount of damage of midbrain dopaminergic neurons but had no effect on the behavior. However, chronic PD model mice displayed massive dopaminergic neuron damage, which was accompanied with anxiety-like behavior in addition to motor dysfunction. The chronic PD model is closer to the pathogenesis of human PD.

## Data Availability

The data used to support the findings of this study are available from the corresponding author upon request.
